# Development and field evaluation of the sentinel mosquito arbovirus capture kit (SMACK)

**DOI:** 10.1186/s13071-015-1114-9

**Published:** 2015-10-06

**Authors:** Brian J. Johnson, Tim Kerlin, Sonja Hall-Mendelin, Andrew F. van den Hurk, Giles Cortis, Stephen L. Doggett, Cheryl Toi, Ken Fall, Jamie L. McMahon, Michael Townsend, Scott A. Ritchie

**Affiliations:** College of Public Health, Medical and Veterinary Sciences, James Cook University, McGregor Rd, Cairns, 4878 QLD Australia; Australian Institute of Tropical Health and Medicine, James Cook University, PO Box 6811, Cairns, QLD 4870 Australia; Department of Agriculture, 114 Catalina Crescent, Cairns International Airport, Cairns, QLD 4870 Australia; Public Health Virology, Forensic and Scientific Services, Department of Health, Queensland Government, Archerfield, 4108 Australia; Private Contracting Engineer, Canberra, ACT 2600 Australia; Department of Medical Entomology, Pathology West-ICPMR, Westmead Hospital, Westmead, NSW 2145 Australia; Bioquip Products, Inc., 2321 E Gladwick St., Rancho Dominguez, Compton, CA 90220 USA

**Keywords:** Arboviruses, Sentinel animals, Mosquitoes, Passive Traps, Surveillance

## Abstract

**Background:**

Although sentinel animals are used successfully throughout the world to monitor arbovirus activity, ethical considerations and cross-reactions in serological assays highlight the importance of developing viable alternatives. Here we outline the development of a passive sentinel mosquito arbovirus capture kit (SMACK) that allows for the detection of arboviruses on honey-baited nucleic acid preservation cards (Flinders Technology Associates; FTA®) and has a similar trap efficacy as standard light traps in our trials.

**Methods:**

The trap efficacy of the SMACK was assessed against Centers for Disease Control and Prevention (CDC) miniature light traps (standard and ultraviolet) and the Encephalitis Vector Survey (EVS) trap in a series of Latin square field trials conducted in North Queensland, Australia. The ability of the SMACK to serve as a sentinel arbovirus surveillance tool was assessed in comparison to Passive Box Traps (PBT) during the 2014 wet season in the Cairns, Australia region and individually in the remote Northern Peninsula Area (NPA) of Australia during the 2015 wet season.

**Results:**

The SMACK caught comparable numbers of mosquitoes to both CDC light traps (mean capture ratio 0.86: 1) and consistently outperformed the EVS trap (mean capture ratio 2.28: 1) when CO_2_ was supplied by either a gas cylinder (500 ml/min) or dry ice (1 kg). During the 2014 arbovirus survey, the SMACK captured significantly (*t*_6_ = 2.1, *P* = 0.04) more mosquitoes than the PBT, and 2 and 1 FTA® cards were positive for Ross River virus and Barmah Forest virus, respectively, while no arboviruses were detected from PBTs. Arbovirus activity was detected at all three surveillance sites during the NPA survey in 2015 and ca. 27 % of FTA® cards tested positive for either Murray Valley encephalitis virus (2 detections), West Nile virus (Kunjin subtype; 13 detections), or both viruses on two occasions.

**Conclusions:**

These results demonstrate that the SMACK is a versatile, simple, and effective passive arbovirus surveillance tool that may also be used as a traditional overnight mosquito trap and has the potential to become a practical substitute for sentinel animal programs.

## Background

Sentinel animals have long been used throughout the world to monitor arbovirus activity and have been employed in Australia since the late 1960s [1–3]. Many Australian states/jurisdictions continue to use sentinel chickens to detect Murray Valley encephalitis virus (MVEV) and West Nile virus (Kunjin subtype; WNV_KUN_), while sentinel pigs have been employed to monitor Japanese encephalitis virus in the Torres Strait and the Cape York Peninsula (JEV) [4, 5]. Despite the ability of sentinel animals to detect arbovirus activity, there are many difficulties with their use. For instance, there are ethical and logistical implications associated with using the animals, as well as challenges in detecting closely related viruses due to cross-reactions in serological assays and a limited ability to only target viruses that infect the selected sentinel animal or those that are transmitted by vectors that feed on the sentinel animals [6, 7]. Given these limitations, a strategy has been developed using CO_2_-baited mosquito traps that house sugar-soaked nucleic preservation cards [6]. While an infected mosquito probes during sugar-feeding virus is expectorated onto the cards, which are subsequently tested for the presence of viral RNA using molecular assays.

To overcome issues with powered traps, such as component malfunction and requirement for electricity to power trap fans and lights, a CO_2_-baited passive (non-battery powered) box trap (PBT) was developed by Ritchie et al. [8]. The PBTs were used to house the honey-soaked cards in a field trial in northern Australia, where multiple arboviruses were detected [9]. Based on the the McPhail fly trap [10] the PBT does not rely on battery-powered fans to capture host-seeking females but instead simply utilises the attractiveness of CO_2_ and the passive retention of captured mosquitoes within a translucent plastic crate. Despite the utility of the PBT as a cheap and efficient passive surveillance device, it may underperform when compared against standard light traps [11] and there are still design principles in need of improvement for long-term deployment. For instance, field trials suggest that keeping mosquitoes alive for several days post capture can increase detection of viruses on the cards [12].

In the current paper, we describe the development and field evaluation of the Sentinel Mosquito Arbovirus Capture Kit (SMACK). The SMACK consists of a CO_2_-baited passive trap that improves upon the original PBT design, particularly modifications to enhance mosquito collection efficacy, mosquito survival post capture, and increased sugar-feeding on honey-baited nucleic acid preservation cards. We then assess the efficacy of the SMACK as a mosquito surveillance device against three standard battery-powered light traps. Finally, we assessed the efficacy of the SMACK for use as a sentinel arbovirus surveillance tool against an unmodified PBT during the 2014 wet season and when deployed as a surveillance tool in the remote Northern Peninsula Area (NPA) of Australia during the 2015 wet season.

## Methods

### General trap design

**Fig. 1:**
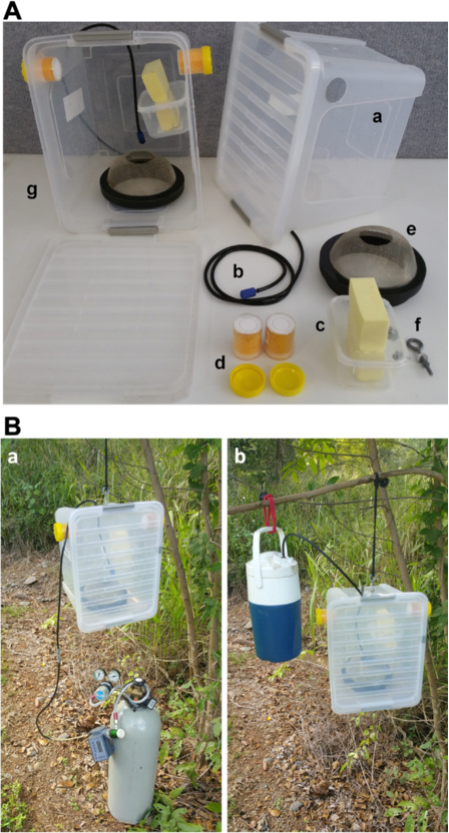
Individual components of the SMACK and field settings with different CO_2_ sources (10 kg cylinder and 1 kg dry ice). **a** The individual components of the SMACK: (*a*) 20 L clear plastic tub with lid, (*b*) 5 mm gas tubing with air stone, (*c*) 500 ml water reservoir and chamois sponge, (*d*) 70 ml FTA® card holders with exposed FTA® cards visible on top, (*e*) 18 cm sieve entry, (*f*) eye bolt from which the trap is hung, and (*g*) a fully assembled trap. **b** The SMACK set in the field with CO_2_ supplied by a 10 kg cylinder with a timer-regulator setup (*a*) and when CO_2_ supplied from 1 kg of dry ice released from an insulated (*b*) cooler

A 20 L translucent plastic storage box (29 × 37, 27 cm deep) (Icon Plastics, Victoria, AU) with a clip-on lid was chosen as the main body of the SMACK, as this size was previously shown to outperform smaller PBT designs [8] (Fig. 1a). Flinders Technology Associates (FTA®) cards (Whatman International Ltd, Maidstone, UK) were placed in 70 ml collection jars in which the bottom had been cut so that the FTA® card was exposed to the mosquitoes. To reduce the desiccation of the honey solution on the FTA® cards, the collection jars contained sponge material (DTA Australia, Victoria, AU) that was soaked in 50 % honey (diluted in distilled water) immediately before the FTA® card was added. This method also allows the cards to be easily inserted and removed from the trap without having to access the cards from inside the trap by anesthetizing captured mosquitoes. The PVC ventilation pipe on top of the PBT described by Ritchie et al. [8] was omitted in the SMACK design and instead a 10 mm hole was created to enable rubber gas tubing to be inserted through the top of the trap. A 12 x 25 mm air stone (Aqua Nova, Petras Fisheries Pty Ltd, Sydney, AU) was attached to the end of the tubing inside the trap to disperse CO_2_ over the sieve. A water reservoir was attached to the inside of the trap and consisted of a 500 ml plastic container (Tellfresh®, Victoria, AU) in which a 3 x 10 cm opening was cut into the lid. The reservoir was filled with distilled water and a chamois sponge (Slurpex, Reedman Agencies, North Sydney, AU) was placed through the opening. A removable 18 cm diameter sieve (2 mm aperture metal mesh) with a 5 cm diameter opening was chosen as the optimal entry configuration. We outline the selection of this entry configuration over that used in the PBT [8] and a smaller mesh sieve below.

### Trap entry comparisons

Three different passive trap entry configurations were compared based on the number of mosquitoes collected per trap night. We used the standard 10 cm hard PVC spigot used in the original PBT, and fine mesh sieves (2 mm aperture metal mesh) of 10 and 18 cm diameters. The mesh sieves were positioned such that the bottom of each faced the inside of the trap and a 5 cm diameter opening was cut in the middle to create an entry point. The field study incorporated a 3 × 3 Latin square experimental design and was conducted in a mixed *Melaleuca* and mangrove swamp adjacent to the Smithfield Waste Disposal Facility near Cairns, Australia (−16.826613°, 145.707065°). All traps were placed approximately 50 m from each other at three different sampling points. The traps were operated for 12 hr each night from 18:00–06:00 with CO_2_ gas supplied at a rate of 500 ml/min using a customized gas regulator (Cortis, unpublished) and timer (Pope #1010371, Toro Australia Pty Ltd) setup (Fig. 1b). All traps were rotated to the next position after each collection to reduce sampling point specific differences. Collected mosquitoes were killed in a freezer and morphologically identified in the laboratory [13]. Two morphologically similar species, *Culex annulirostris* (Skuse) and *Culex sitiens* (Wiedemann)*,* were grouped together as the *Cx. sitiens* subgroup because morphological differentiation is difficult in cases where key diagnostic features are damaged [14]. Differences in the mean number of mosquitoes collected were compared by analysis of variance (ANOVA) on log (n + 1) transformed abundance data. The effect of entry type and trap location (i.e. Latin square number) on recorded community composition (relative abundance of each species) was analysed by permutational multivariate analysis of variance (PerMANOVA) models of Bray-Curtis dissimilarities [15].

### Comparison to standard mosquito light traps

Following the determination of the entry to be used in the SMACK we conduced Latin square trials to assess the efficacy of the SMACK as a mosquito surveillance device compared to a CDC model 512 miniature light trap (CDC, John W Hock; http://johnwhock.com), CDC model 912 Miniature Downdraft Blacklight (CDC + UV, John W. Hock), and an Encephalitis Vector Survey (EVS) light trap (Australian Entomological Supplies; http://www.entosupplies.com.au). Two full 4x4 Latin square trials were conducted, one in which CO_2_ was only supplied using 1 kg dry ice and one in which CO_2_ was only supplied through a 10 kg compressed gas cylinder at 500 ml/min (Fig. 1b). When baiting passive traps with dry ice, it is critical that the CO_2_ gas hose is attached to the top of the insulated cooler (Fig. 1b). Our experience indicates that hoses attached to the bottom of the cooler can become clogged from ice forming from water that condenses within the hose/cooler junction. In addition to recording the total number of mosquitoes collected by each trap type, we also recorded the total number of non-target insects, or by-catch, collected for each trap type. Mosquitoes were identified to species, whereas non-target insects were identified to order. The nightly trapping regime, location of the study, and statistical analyses were the same as those outlined in the entry type comparisons above.

### Mosquito survival study

To assess the benefits of the addition of the water reservoir and chamois in the SMACK on mosquito survival (longevity) we analysed daily survivorship between SMACKs containing water reservoirs and unmodified PBTs that do not contain such reservoirs. Two traps of each type were stocked with ca. 200 field collected mosquitoes, primarily a mixture of *Aedes vigilax* (Skuse)*, Cx. annulirostris,* and *Verrallina funerea* (Theobald). The mosquitoes were collected the previous evening and were returned to the laboratory where they were then distributed amongst the traps. The traps were hung outside the Mosquito Research Facility on the James Cook University (Cairns) campus beneath a 99 % shade cloth. Each trap contained temperature and humidity loggers (iButtonLink LLC, Whitewater, WI, USA) and were suspended a distance of 1 m from the ground. An additional pair of data loggers was placed underneath a table adjacent to the hanging traps to monitor ambient air temperature and humidity. Temperature and humidity recordings were taken every 15 min for the length of the study. The number of dead mosquitoes was recorded each day for a period of 14 d. Differences in daily survivorship, as determined by Kaplan-Meier survivorship curves, between the SMACKs and PBTs were determined by the log-rank test [16].

### Mosquito sugar-feeding rate

**Fig. 2:**
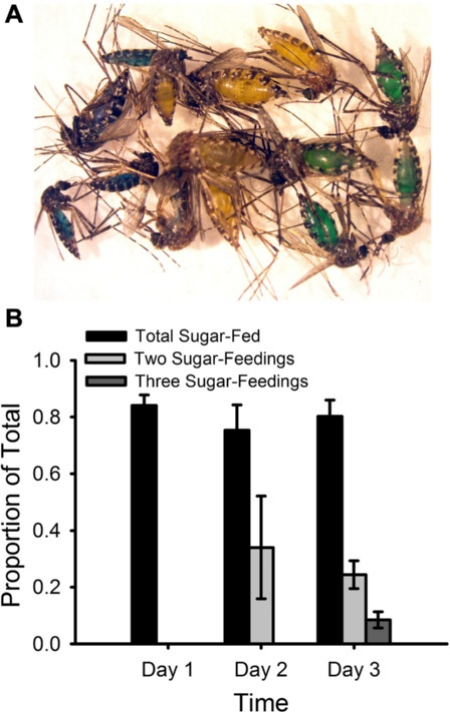
Examples of recently ingested sugar meals and daily mosquito sugar-feeding rates on honey-baited FTA® cards. **a** Recent sugar meals easily observable in the abdomens of field-collected mosquitoes exposed to differently dyed honey-soaked FTA® cards. The figure depicts individual sugar-feedings on either blue (day 1) or yellow (day 2) dyed honey and the generation of a green colour if a second sugar-feeding occurred during day 2. **b** Daily sugar-feed rates (mean ± SE) of females on honey-baited FTA® cards when housed in SMACKs for a period of 3 d

We used food colouring added to the honey [6] prior to application on the FTA® cards to quantify the sugar-feeding rate of field-collected mosquitoes housed in SMACKs for a period of 3 d. Two SMACKs were stocked with ca. 500 female mosquitoes, primarily a mixture of *Ae. vigilax, Cx. annulirostris,* and *Ve. funerea*, which were collected the previous evening in the field. Each morning for a period of 3 days different coloured (day 1, blue; day 2, yellow; day 3, red) honey-soaked FTA® cards were introduced into each trap. The following morning a sub-sample of 100 mosquitoes was aspirated from each trap and the sugar-feeding status of each female was determined by observing the abdomen through a stereo microscope. If the female had sugar-fed the colour of the dye on the FTA® card was easily observed within the abdomen (Fig. 2a). After determining the feeding status of each female they were removed from the study. For each consecutive day, the number of secondary sugar-meals was determined based on the mixing of the two different coloured dyes (e.g. blue and yellow = green). On the third day, the number of third sugar-feedings was determined by the mixing of the three different dyes (i.e. purple/brown).

### Virus detection in SMACK vs PBT

The ability of the SMACK to detect endemic arboviruses in a field setting was assessed against an unmodified PBT during a pilot trial in May, 2014. The study was conducted in the mixed *Melaleuca* and mangrove swamp in which the trap comparison studies were performed and in a tropical rainforest site (−16.818193°, 145.680896°) adjacent to the James Cook University, Cairns, Australia, campus. One of each trap type was set at each location and the traps were set a distance of 50 m apart within each site. Since this study began prior to the optimization of the chamois water reservoir, the pilot SMACK was set with two 8 x 13 cm thin sponge panels that were moistened with water then fixed to the trap walls with a screw. Traps were operated on a weekly collection schedule during which CO_2_ gas was released nightly from 18:00–06:00 at 500 ml/min. Each trap was set with two honey-soaked FTA® cards. At the end of each weekly collection period the FTA® cards were collected and replaced, the sponges rewetted, and captured mosquitoes removed. Captured mosquitoes were returned to the laboratory and morphologically identified [13]. Collected FTA® cards were individually wrapped in Glad Snap Lock Mini® bags (Clorox Australia Pty Ltd, Padstow, NSW), labeled and transported to the Centre for Infectious Diseases and Microbiology Laboratory Services (CIDMLS), Institute for Clinical Pathology and Medical Research (ICPMR), Westmead Hospital, where they were stored at −80 °C until processed for virus detection. The cards were screened for the presence of Ross River virus (RRV) and Barmah Forest virus (BFV) RNA, the two most common and widespread arboviruses in Australia [17], as well as WNV_KUN_ and MVE by a nested real-time RT-PCR using EvaGreen (Biotium, Hayward, CA) following published protocols [12]. Positive RRV and BFV samples were confirmed by sequencing the product of a section of nonstructural protein P4 (174 bp and 219 bp, respectively for RRV and BFV) and comparing generated sequences to GenBank accession numbers [GQ433354.1; RRV] and [AF339488.1; BFV].

### Sentinel arbovirus surveillance study

A longitudinal study was conducted in the remote Northern Peninsula Area (NPA) of northern Australia to assess the ability of the SMACK to serve as a sentinel mosquito arbovirus detection system under natural field conditions. The NPA area was chosen based on its remoteness and historic use of sentinel pigs to monitor Japanese encephalitis virus (JEV) activity [18, 19]. The field sites included the NPA waste disposal site located in Bamaga (−10.893184°, 142.395665°), a cattle yard in Seisia (−10.852551°, 142.370513°), and a piggery 3 km from Injinoo (−10.891992°, 142.353362°). A single SMACK was set at each site during the first week of February 2015, and operated on a fortnightly collection schedule until May 11^th^ 2015. Each trap contained two honey-soaked FTA® cards and CO_2_ gas was supplied through a 10 kg gas cylinder and regulated to operate for 12 hr each night (18:00–06:00) at a rate of 500 ml/min. At the end of each fortnightly collection period, the traps were reset by discarding dead mosquitoes, refilling the water reservoir, and replacing the honey-soaked FTA® cards with new cards. Collected FTA® cards were wrapped in Parafilm M® (Bemis NA, Neenah, WI), labelled, and posted to QHFSS laboratories for analysis. FTA® cards were processed for MVEV, WNV_KUN_ and JEV virus detection following established protocols [6, 9].

## Results

### Trap entry comparisons

**Fig. 3:**
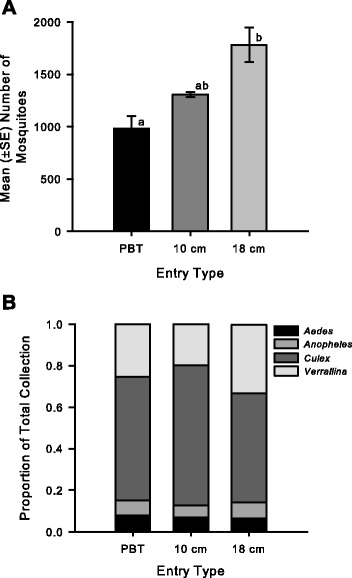
Mean number of mosquitoes collected per entry type per night and collection composition summarized by genera. **a** Mean (±SE) number of mosquitoes collected by each entry type (PVC spigot, 10 cm and18 cm diameter mesh sieve with a 5 cm diameter entry hole) per trap night and **b** the mean proportion of each nightly collection total comprised of mosquitoes species belonging to four different mosquito genera (*Aedes*, *Anopheles*, *Culex*, and *Verrallina*). Different letters indicate a significant (*P-value* < 0.05) difference (ANOVA, Tukey HSD post-hoc analysis)

Significant differences (*F*_2,6_ = 11.4, *P =* 0.02) were observed among the entry types in the number of mosquitoes collected per trap night (Fig. 3a). Overall, the 18 cm diameter (mean ± SE mosquitoes captured = 1781 ± 165) mesh sieve outperformed the PVC-Spigot (980 ± 121) of the PBT and the smaller 10 cm sieve (1307 ± 23). No significant difference (*F*_2,4_ = 4.5, *P* = 0.91) in the composition of mosquito collections (i.e. the proportion of each collection total belonging to a particular genus) was observed among the entry types (Fig. 3b). Based on these results, the 18 cm diameter sieve was chosen as the entry configuration for the SMACK.

### Comparison to standard mosquito light traps

**Fig. 4:**
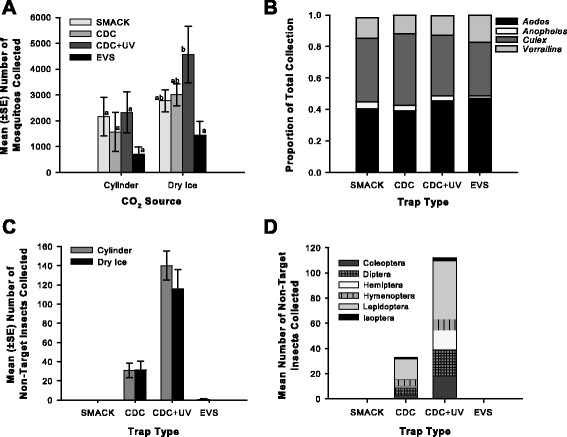
Mean nightly mosquito collections, trap composition, and non-target organisms collected per trap per night, **a** Nightly collection totals (mean ± SE) by trap type across the two CO_2_ sources (gas cylinder at 500 ml/min and 1 kg dry ice). **b** Proportion of each collection total comprised of mosquitoes species belonging to four different mosquito genera (*Aedes, Anopheles, Culex,* and *Verrallina*). **c** Mean (±SE) of non-target insects collected per trap night for each trap type across the two CO_2_ sources (gas cylinder at 500 ml/min and 1 kg dry ice). **d** Mean number of non-target insects collected per trap night for each trap averaged across both CO_2_ treatments and summarized by order. Different letters indicate a significant (*P-value* < 0.05) difference (ANOVA, Tukey HSD post-hoc analysis)

The SMACK collected comparable numbers as both CDC trap designs and the EVS trap when CO_2_ was supplied either with dry ice or via compressed gas (Fig. 4a, Table 1). Species richness (ca. mean of 8 species/trap for each trial) and community composition (Fig. 4b) was comparable among all trap types when CO_2_ was supplied from dry ice or via compressed gas. The light traps, especially the CDC + UV captured significantly (*P <* 0.001) more non-target insects (Fig. 4c) than the SMACK and EVS traps. Throughout the 8 trappings, the SMACK collected no non-target insects. The majority of non-target insects belonged to the orders Lepidoptera, Diptera, Coleoptera, and Hymenoptera (Fig. 4d).

**Table 1:** Mean (±SE) species abundance for each trap type when CO_2_ was supplied by a gas cylinder at 500 ml/min or with 1 kg of dry ice pellets

	Sentinel Arbovirus Capture Kit (SMACK)		CDC model 512 miniature light trap (incandescent)		CDC model 912 miniature light trap (ultraviolet)		Encephalitis Vector Survey trap	
	Cylinder	Dry Ice	Cylinder	Dry Ice	Cylinder	Dry Ice	Cylinder	Dry Ice
*Aedes alboscutellatus*	2 (3)	0	0	0	0	0	0	0
*Aedes alternans*	1 (1)	10 (10)	11 (7)	0	4 (4)	31 (19)	3 (2)	7 (8)
*Aedes kochi*	181 (131)	74 (49)	62 (41)	61 (27)	57 (23)	106 (59)	92 (37)	63 (46)
*Aedes notoscriptus*	20 (17)	13 (8)	15 (9)	8 (5)	11 (4)	0	13 (2)	2 (3)
*Aedes palmarum*	0	9 (6)	0	0	0	0	0	0
*Aedes tremulus*	0	0	0	4 (5)	0	8 (8)	0	0
*Aedes vigilax*	198 (43)	1597 (362)	82 (31)	1941 (295)	336 (105)	3198 (906)	53 (16)	998 (431)
*Anopheles bancroftii*	0	0	2 (2)	0	0	9 (9)	0	3 (3)
*Anopheles farauti* sensu lato	97 (60)	131 (11)	58 (24)	93 (29)	84 (41)	158 (46)	9 (3)	27 (14)
*Coquillettidia crassipes*	0	0	0	0	4 (4)	0	0	0
*Coquillettidia xanthogaster*	0	0	0	0	0	0	0	1 (2)
*Culex cubiculi*	0	0	0	0	0	6 (6)	0	12 (5)
*Culex gelidus*	1 (1)	0	0	0	0	27 (0)	0	0
*Culex hilli*	0	50 (8)	11 (5)	31 (14)	0	37 (21)	2 (1)	26 (9)
*Culex pullus*	3 (2)	0	1 (2)	0	16 (14)	4 (5)	1 (5)	0
*Culex sitiens* subgroup	1229 (524)	686 (146)	1176 (513)	639 (123)	1372 (514)	862 (239)	425 (193)	221 (102)
*Mansonia septempunctata*	0	8 (5)	0	0	0	0	1 (2)	0
*Mansonia uniformis*	0	13 (8)	6 (7)	2 (2)	0	7 (5)	0	2 (3)
*Tripteroides magnesianus*	9 (9)	0	0	0	0	0	0	0
*Uranotaenia* sp.	2 (3)	0	0	4 (5)	6 (4)	7 (7)	0	0
*Verrallina carmenti*	99 (44)	42 (26)	85 (75)	56 (33)	134 (58)	41 (30)	34 (15)	8 (7)
*Verrallina funerea*	316 (99)	125 (28)	192 (39)	158 (58)	407 (115)	78 (38)	184 (52)	74 (27)
*Verrallina lineata*	0	6 (6)	1 (2)	0	0	0	0	0

### Mosquito survival study

**Fig. 5:**
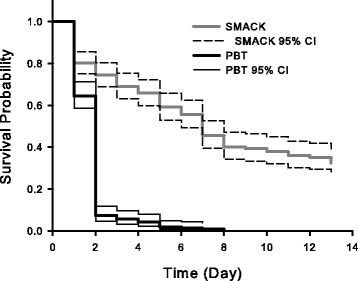
Daily survival of field-collected mosquitoes housed in a SMACK and unmodified PBT over 14 days. Daily survival probability (mean and 95 % confidence intervals) of field-collected mosquitoes housed in a SMACK and unmodified PBT for a period of 14 days

The addition of the chamois sponge and water reservoir resulted in significant increases in daily relative humidity (*t*_12_ *=* 2.68, *P =* 0.01), with a mean (± SE) daily increase of 7.0 ± 1.3 % compared to the control PBT, which did not contain the water reservoir (Table 2). No difference in the relative humidity recorded inside the PBT and ambient relative humidity was observed. No significant difference in daily high temperatures recorded within the traps was observed, as well as when compared against the ambient daily high temperature recorded each day. The increased humidity in the SMACK corresponded to a significant (*X*_1_ *=* 28.1, *P <* 0.001) increase in the daily survival probability of captured mosquitoes (Fig. 5). Overall, 93.3 ± 1.9 % of mosquitoes in control traps were dead after 2 days, whereas in traps with sponge, 47.0 ± 7.6 % and 33.8 ± 6.5 % were alive after 7 and 14 days, respectively.

**Table 2:** Summary of daily relative humidity and temperature recordings observed during the mosquito survivorship (longevity) study

Relative Humidity (%)			
	Ambient Outside	Inside PBT	Inside SMACK
Mean (±SE)	81.0 (2.4)	80.3 (2.5)	88.0 (1.4)
Record Low	53.3	53.6	68.9
Record High	99.6	99.2	99.1
Temperature (°C)			
	Ambient Outside	Inside PBT	Inside SMACK
Mean (±SE)	27.5 (0.41)	27.9 (0.45)	27.7 (0.44)
Record Low	24.2	24.3	24.2
Record High	31.5	32.4	32.1

Summary of daily relative humidity and temperature recordings observed during the mosquito survivorship (longevity) study. The SMACK contained a water reservoir from which moisture was released from a chamois sponge (Slurpex, Reedman Agencies, North Sydney, AU). PBTs did not contain a water reservoir system. Temperature and humidity recordings were taken every 15 min for the length of the study (14 d)

### Mosquito sugar-feeding rate

The sugar-feeding study revealed that a mean (± SE) of 80.0 ± 6.1 % of field-collected mosquitoes were sugar-fed on any given day (Fig. 2b) and that 29.2 ± 11.5 % had sugar-fed at least twice between days 2 and 3. Further, 8.5 ± 2.9 % of field-collected mosquitoes had sugar-fed a minimum of three times by the end of day 3. No significant difference (*F*_1,3_ = 1.3, *P* = 0.89) in sugar-feeding rates among the species used in the study was observed.

### Virus detection in SMACK vs. PBT

The pilot SMACK captured significantly more mosquitoes than the PBT. A mean (± SD) of 1298 ± 1381 and 555 ± 892 mosquitoes were captured in seven weekly paired trappings of the SMACK and the PBT, respectively *(t*_6_ = 2.02, *P* = 0.046). A total of 12 FTA® cards each for the SMACK and PBT were tested for the presence of BFV and RRV. Two FTA® card pools (2 cards/pool) collected from SMACKs were positive, both of which originated from the tropical rainforest habitat. One pool tested positive for RRV and the other pool tested positive for BFV and RRV. In contrast, no FTA® card pools were positive from the unmodified PBT.

### Sentinel arbovirus surveillance study

A total of 48 individual FTA® cards, 16 from each trap site, were tested for the presence of MVEV, JEV and WNV_KUN_ (Table 3). Overall, 13 FTA® cards were positive, comprising 13 cards positive for WNV_KUN_ and 2 cards positive for both WNV_KUN_ and MVEV. Arbovirus detections were greatest during the month of March (7 positive cards) and at the NPA waste disposal site (5 positive cards resulting in 7 virus detections). WNV_KUN_ was the most widely distributed arbovirus (detected multiple times at all three collection sites), whereas MVEV had a limited distribution (detected only at the NPA waste disposal site) and was only detected during the month of March.

**Table 3:** Summary of fortnightly arbovirus surveillance study conducted in the Northern Peninsula Area (NPA) of Queensland, Australia

MONTH	LOCATION	NO. CARDS TESTED	NO. POS	MVEV POS	WNV_KUN_ POS
February	NPA WDS, Bamaga	4	0	0	0
	Cattle Yard, Seisia	4	0	0	0
	Rocky Piggery, Injinoo	4	0	0	0
March	NPA WDS, Bamaga	6	3	2	3
	Cattle Yard, Seisia	6	2	0	2
	Rocky Piggery, Injinoo	6	2	0	2
April	NPA WDS, Bamaga	4	2	0	2
	Cattle Yard, Seisia	4	2	0	2
	Rocky Piggery, Injinoo	4	2	0	2
May	NPA WDS, Bamaga	2	0	0	0
	Cattle Yard, Seisia	2	0	0	0
	Rocky Piggery, Injinoo	2	0	0	0
	Total	48	13	2	13

Summary of fortnightly arbovirus surveillance study conducted in the Northern Peninsula Area (NPA) of Queensland, Australia using honey-soaked FTA® cards housed in the sentinel mosquito arbovirus capture kit (SMACK). Arboviruses tested for included Japanese encephalitis virus (JEV), Murray Valley encephalitis virus (MVEV) and West Nile virus (Kunjin subtype) (WNV_KUN_). No FTA® cards tested positive for JEV. Each individual trap was set with two honey-soaked FTA® cards

## Discussion

Effective disease surveillance forms a vital component of any program aimed at reducing the impact of arboviruses on human and animal health. However, the logistics of monitoring arbovirus activity in remote locations using standard battery-powered mosquito traps is often problematic, while cross-reactions in serological assays can greatly reduce the specificity of sentinel animal serology surveys [4]. In the current study, we highlight the development of a sentinel mosquito arbovirus capture kit (SMACK) that does not require battery-power, can compete with standard mosquito traps, maximizes mosquito longevity after collection, and is successful at monitoring arbovirus activity in remote locations.

The enhanced survivorship of field-collected mosquitoes housed in SMACKs compared to those housed in PBTs allow for a maximum number of individual feeding events to occur on FTA® cards. Our results reveal that over a period of 3 days >80 % of field-collected mosquitoes will acquire at least a single sugar-meal, and approximately 20–25 % will have sugar-fed at least twice over the same time period. These results suggest that although mosquitoes do crowd onto honey-baited FTA® cards, crowding does not inhibit sugar feeding. Consequently, previous protocols advising the use of insecticides (added to the honey) to reduce crowding on honey-baited FTA® cards [8] should be amended to maximize the number of feeding events occurring, and subsequently the amount of virus being expectorated upon the cards.

The detection of four different arboviruses, including the two most important arboviruses in Australia, RRV and MVEV, as well as the detection of multiple viruses on a single FTA® card (WNV_KUN_, MVEV), demonstrates that the SMACK is capable of adequately sampling the local vector population to monitor arboviruses vectored by different mosquito species with varying ecologies. While the *Cx. sitiens* subgroup will include both *Cx. sitiens* and *Cx. annulirostris*, which we did not separate in the current study, earlier studies in which large numbers of *Cx. annulirostris* were collected at the same sites [8] and other areas where *Cx. sitiens* does not occur [9] indicate that passive box traps are effective at capturing *Cx. annulirostris* [8]. Furthermore, although each detected arbovirus is primarily vectored by *Cx. annulirostris,* arguably the most medically important mosquito in Australia [20, 21], other vectors of RRV and BFV, such as *Ae. vigilax* [22, 23], accounted for a large proportion of captured mosquitoes. The detection of WNV_KUN_ in 13/48 FTA® cards from SMACKs set at three sites suggests that the method is sensitive for flavivirus detection in remote areas. Further, the detection of MVEV on two FTA® cards from the SMACK set at Bamaga represents the fourth detection of MVEV using passive traps fitted with FTA® cards [24]. These results, in combination with previous studies which suggest sugar-based systems were more sensitive at detecting arbovirus activity than concurrently monitored sentinel animals [9, 25], highlight the potential of sugar-based systems to complement and possibly serve as a substitute for sentinel animal programs. However, such comparisons are still limited and full parallel trials comparing the sensitivity, utility and cost of the SMACK, as well as other sugar-based arbovirus surveillance systems, to sentinel animals are needed before any operational changes are made.

In addition to its utility as a long-term arbovirus surveillance device, the SMACK was equally effective as the CDC miniature light traps (average capture ratio 0.86: 1), and more effective than the EVS trap (capture ratio 2.28: 1), at monitoring local mosquito populations when operated on a nightly collection schedule. Further, no difference in the composition of mosquito collections was observed between the SMACK and light traps indicating that there was little bias in terms of the species collected. Additionally, in contrast to the CDC light traps, the SMACK did not collect any non-target insects during field testing resulting in decreased processing times relative to the CDC light traps. These results, combined with its lack of reliance on battery or mains power (household/city power), make the SMACK a potential inexpensive substitute for traditional mosquito traps that are deployed for a single night. We note that variations in environmental conditions and differences in the responses of individual mosquito species can dramatically influence individual trap efficacy [26, 27]. This was evident in field comparisons of the PBT and EVS trap undertaken in different regions of Australia in which the EVS trap substantially outperformed the PBT [11]. Accordingly, potential SMACK users should initially run this trap in parallel with existing systems to check for relative sensitivities at collecting mosquitoes and costs of operation before changes to existing operational protocols are made. Potential users should consider other practicalities such as cost per unit and size when considering using the SMACK as an overnight mosquito trap. For instance, the SMACK will be available commercially for ca. 80.00 USD (bioquip.com), which makes it the cheapest of the traps tested (CDC, $106.00 USD, johnwhock.com; CDC + UV, $169.00 USD, johnwhock.com; EVS, $96.95 USD, bioquip.com), while the larger size of the SMACK may be cumbersome during large scale overnight surveillance operations when laboratory and transport space is limited.

## Conclusions

These results demonstrate that the SMACK has the potential to be a versatile, simple, and highly sensitive arbovirus surveillance tool that may also be used as a traditional overnight mosquito trap. The versatility of the SMACK enables it to be used to complement existing sentinel animal programs and, importantly, serve as a viable substitute when the use of sentinel animals is not feasible. There is also the potential to detect additional vector-borne pathogens transmitted by other hematophagous arthropods using the SMACK. For instance, sugar-baited FTA® cards have been used to detect Schmallenberg virus in the expectorate of *Culicoides* biting midges [28], which are traditionally surveyed using CO_2_-baited traps [29, 30]. Finally, the efficacy and simplicity of the SMACK make it suitable for use in developing countries in which the need for cheap, simple, and efficient arbovirus surveillance tools is often greatest.

## References

[1] George TS (1980). A sentinel herd system for the study of arbovirus infections in Australia and Papua-New Guinea. Vet Res Commun.

[2] Monath T, Sabattini M, Pauli R, Daffner J, Mitchell C, Bowen G (1985). Arbovirus investigations in Argentina, 1977–1980. IV. Serologic surveys and sentinel equine program. Am J Trop Med Hyg.

[3] Komar N (2001). West Nile virus surveillance using sentinel birds. Ann N Y Acad Sci.

[4] van den Hurk AF, Hall-Mendelin S, Johansen CA, Warrilow D, Ritchie SA. Evolution of mosquito-based arbovirus surveillance systems in Australia. BioMed Research International 2012, 2012. doi: 10.1155/2012/325659. 10.1155/2012/325659 PMC3312405 22505808

[5] Knope K, Whelan P, Smith D, Nicholson J, Moran R, Doggett S (2013). Arboviral diseases and malaria in Australia, 2010–11: annual report of the National Arbovirus and Malaria Advisory Committee. Commun Dis Intell Q Rep.

[6] Hall-Mendelin S, Ritchie SA, Johansen CA, Zborowski P, Cortis G, Dandridge S (2010). Exploiting mosquito sugar feeding to detect mosquito-borne pathogens. Proc Natl Acad Sci.

[7] Hall RA, Blitvich BJ, Johansen CA, Blacksell SD. Advances in arbovirus surveillance, detection and diagnosis. BioMed Research International 2012, 2012. doi:10.1155/2012/512969. 10.1155/2012/512969 PMC3363164 22665984

[8] Ritchie SA, Cortis G, Paton C, Townsend M, Shroyer D, Zborowski P (2013). A simple non-powered passive trap for the collection of mosquitoes for arbovirus surveillance. J Med Entomol.

[9] van den Hurk AF, Hall-Mendelin S, Townsend M, Kurucz N, Edwards J, Ehlers G (2014). Applications of a sugar-based surveillance system to track arboviruses in wild mosquito populations. Vector-Borne Zoonotic Dis.

[10] Thomas DB, Holler TC, Heath RR, Salinas EJ, Moses AL (2001). Trap-lure combinations for surveillance of Anastrepha fruit flies (Diptera: Tephritidae). Fla Entomol.

[11] Doggett S, Clancy J, Haniotis J, Russell RC, McIntyre L, Lim H (2014). The NSW Arbovirus Surveillance and Mosquito Monitoring Program, 2013–2014.

[12] Flies EJ, Toi C, Weinstein P, Doggett SL, Williams CR (2015). Converting mosquito surveillance to arbovirus surveillance with honey-baited nucleic acid preservation cards. Vector-Borne Zoonotic Dis.

[13] Ehlers G, Alsemgeest D. Common mosquitoes of North Queensland: identification and biology of adult mosquitoes. Kirwan, QLD, 4817 AU: Mosquito Control Association of Australia Inc. 2010.

[14] Jansen CC, Hemmerter S, Hurk AF, Whelan PI, Beebe NW (2013). Morphological versus molecular identification of Culex annulirostris Skuse and Culex palpalis Taylor: key members of the Culex sitiens (Diptera: Culicidae) subgroup in Australasia. Aust J Entomol.

[15] Anderson MJ (2001). A new method for non‐parametric multivariate analysis of variance. Austral Ecol.

[16] Keven JB, Henry-Halldin CN, Thomsen EK, Mueller I, Siba PM, Zimmerman PA (2010). Pyrethroid susceptibility in natural populations of the Anopheles punctulatus group (Diptera: Culicidae) in Papua New Guinea. Am J Trop Med Hyg.

[17] Kelly‐Hope LA, Kay BH, Purdie DM, Williams GM (2002). Infectious disease: the risk of Ross River and Barmah Forest virus disease in Queensland: implications for New Zealand. Aust N Z J Public Health.

[18] Van Den Hurk AF, Montgomery BL, Northill JA, Smith IL, Zborowski P, Ritchie SA (2006). The first isolation of Japanese encephalitis virus from mosquitoes collected from mainland Australia. Am J Trop Med Hyg.

[19] Ritchie SA, Van Den Hurk AF, Zborowski P, Kerlin TJ, Banks D, Walker JA (2007). Operational trials of remote mosquito trap systems for Japanese encephalitis virus surveillance in the Torres Strait. Aust Vector-Borne Zoonotic Dis.

[20] Harley D, Sleigh A, Ritchie S (2001). Ross River virus transmission, infection, and disease: a cross-disciplinary review. Clin Microbiol Rev.

[21] Kay B, Fanning I, Carley J (1984). The vector competence of Australian Culex annulirostris with Murray Valley encephalitis and Kunjin viruses. Aust J Exp Biol Med Sci.

[22] Russell RC (2002). Ross River virus: ecology and distribution. Annu Rev Entomol.

[23] Boyd AM, Kay BH (1999). Experimental infection and transmission of Barmah Forest virus by Aedes vigilax (Diptera: Culicidae). J Med Entomol.

[24] Kurucz N, Wenham J, Hunt N, Melville L (2014). Murray Valley encephalitis virus detection using honeybait cards in the Northern Territory in 2013. Mosqu Bites.

[25] Lothrop HD, Wheeler SS, Fang Y, Reisen WK (2012). Use of scented sugar bait stations to track mosquito-borne arbovirus transmission in California. J Med Entomol.

[26] Silver JB. Sampling adults with visual attraction traps, sound traps and other miscellaneous attraction traps. Mosqu Ecol Field Sampling Methods. 2008;1027–1048.

[27] Brown HE, Paladini M, Cook RA, Kline D, Barnard D, Fish D (2008). Effectiveness of mosquito traps in measuring species abundance and composition. J Med Entomol.

[28] Veronesi E, Henstock M, Gubbins S, Batten C, Manley R, Barber J (2013). Implicating Culicoides biting midges as vectors of Schmallenberg virus using semi-quantitative RT-PCR. PLoS ONE.

[29] Ritchie SA, Van Essen PHA, Kemme JA, Kay BH, Allaway D (1994). Response of biting midges (Diptera: Ceratopogonidae) to carbon dioxide, octenol, and light in southeastern Queensland. Australia J Med Entomol.

[30] Takken W, Verhulst N, Scholte E-J, Jacobs F, Jongema Y, van Lammeren R (2008). The phenology and population dynamics of Culicoides spp. in different ecosystems in The Netherlands. Prev Vet Med.

